# A phase II study of a 5T4 oncofoetal antigen tumour-targeted superantigen (ABR-214936) therapy in patients with advanced renal cell carcinoma

**DOI:** 10.1038/sj.bjc.6603567

**Published:** 2007-02-06

**Authors:** D M Shaw, N B Connolly, P M Patel, S Kilany, G Hedlund, Ö Nordle, G Forsberg, J Zweit, P L Stern, R E Hawkins

**Affiliations:** 1 Paterson Institute for Cancer Research, Christie Hospital NHS Trust, Manchester M20 4BX, UK; 2 Christie Hospital NHS Trust, Manchester, M20 4BX, UK; 3 St James University Hospital, Leeds LS9 7TF, UK; 4 Active Biotech AB, Scleelevagen 22, Lund SE-220 07, Sweden

**Keywords:** tumour-targeted superantigen, tumour-associated antigen, renal cell carcinoma, Motzer

## Abstract

In a phase II study, 43 renal cell carcinoma patients were treated with individualised doses of ABR-214936; a fusion of a Fab recognising the antigen 5T4, and Staphylococcal enterotoxin A. Drug was given intravenously on 4 consecutive days, treatment was repeated 1 month later. Treatment was associated with moderate fever and nausea, but well tolerated. Of 40 evaluable patients, 28 had disease control at 2 months, and at 4 months, one patient showed partial response (PR) and 16 patients stable disease. Median survival, with minimum follow-up of 26 months was 19.7 months with 13 patients alive to date. Stratification by the Motzer's prognostic criteria highlights prolonged survival compared to published expectation. Patients receiving higher drug exposure had greater disease control and lived almost twice as long as expected, whereas the low-exposure patients survived as expected. Sustained interleukin-2 (IL-2) production after a repeated injection appears to be a biomarker for clinical effect, as the induced-IL-2 level on the day 2 of treatment correlated with survival. The high degree of disease control and the prolonged survival suggest that this treatment can be effective. These findings will be used in the trial design for the next generation of drug, with reduced antigenicity and toxicity.

Renal tumours account for approximately 200 000 new cases worldwide each year, roughly 3% of all cancers and the incidence is increasing, though this is largely owing to improvements in diagnosis. It often presents at an advanced stage with approximately 1/3 of cases already having established metastases leading to high mortality rates (Quinn *et al*, 2001). Approximately, 1/3 of patients with local disease are cured by surgery but as many as 40% will go on to develop metastases after surgery. Treatment options for metastatic disease are limited, as the cancer tends to be resistant to both chemotherapy and radiotherapy (Mulders *et al*, 1997). Instead, interferon-*α* (IFN-*α*) and interleukin-2 (IL-2) are widely used for the treatment of metastatic renal cell carcinoma (RCC). However, response rates are low (14–20%) with some survival advantage demonstrated for IFN-*α*, but not for IL-2 (Collaborators, 1999; Yang *et al*, 2003). Thus, so far immunotherapy has been the most useful therapeutic approach for RCC, but recently antiangiogenic agents have shown encouraging data in clinical trials (de Gramont and Van Cutsem, 2005; Rini *et al*, 2005; Motzer *et al*, 2006).

Tumour-targeted superantigens (TTS) work by polyclonal activation of the patients' own T cells. Superantigens such as Staphylococcal enterotoxin A (SEA) are secreted molecules employed by pathogens to evade the immune system. Their binding to major histocompatibility complex (MHC) class II molecules on antigen presenting cells (APC) and T-cell receptors of a subset of T cells-bearing particular V*β*-chains (Dermime *et al*, 2004; Petersson *et al*, 2004) activates the T cells in an antigen-independent manner. Activation leads to the expression of perforin and the production of cytotoxic and proinflammatory cytokines and thus death of the APC (Fischer *et al*, 1990; Dohlsten *et al*, 1991b). Targeting of superantigens towards tumours induces a strong, local cytotoxic T-cell attack, which directly kills tumour cells and leads to inflammation and the local accumulation of tumouricidal cytokines (Dohlsten *et al*, 1991a, 1994, 1995; Litton *et al*, 1997, 1999).

ABR-214936 is a recombinant fusion protein between a modified form of SEA and a murine Fab that recognises the tumour-associated antigen 5T4. In this, SEA has been engineered for reduced MHC class II binding giving it a much improved safety profile compared to fusions with wild-type SEA (Alpaugh *et al*, 1998a; Brodin *et al*, 1998; Forsberg *et al*, 2001; Cheng *et al*, 2004). The 5T4 oncofoetal antigen is a transmembrane glycoprotein (Hole and Stern, 1988, 1990). It is expressed by a wide range of cancers (Southall *et al*, 1990), including gastric, colorectal, ovarian (Dermime *et al*, 2004), non-small cell lung (NSCLC) (19) and at high levels by 95% of RCC (Forsberg *et al*, 2001; Griffiths *et al*, 2005). Despite its widespread expression in malignancy, it shows only limited expression in normal adult tissues (Southall *et al*,tvjmline 1990; Forsberg *et al*, 2001), making it an ideal target for antibody-mediated therapy. Renal cell carcinoma is particularly suitable for therapy with ABR-214936 as it is well vascularised and a high proportion (>95%) of tumours are positive for 5T4 expression.

In the phase I study of ABR-214936 in NSCLC, the maximum tolerated dose (MTD) as a function of pre-existing anti-SEA antibody was determined (Cheng *et al*, 2004). The reported major side effects of ABR-214936 included pyrexia, hypotension, rigours, nausea and lethargy. Here we report the further investigation of ABR-214936 at the phase II level in RCC patients using a dose-regimen based on the findings of the earlier study.

## PATIENTS AND METHODS

### Patient population

This open-label phase II study was conducted between December 2001 and March 2003 according to the protocol approved by the Local Research Ethical Committees of both centres. All patients gave informed written consent, were ⩾18 years of age, of either sex with greater than 80% Karnofsky's rating and life expectancy greater than 6 months. Adequate organ function was defined as WBC ⩽3.000 mm^−3^, absolute neutrophil count ⩽1500 mm^−3^ or platelets ⩽100 000 mm^−3^, haemoglobin <100 g l^−1^, bilirubin ⩽2 times the upper normal limit and creatinine 1.5 times normal, FEV_1_>70 and SaO_2_>90%. Patients with uncorrected hypercalcaemia, poorly controlled hypertension or taking *β*-blockers were excluded, as were patients with cardiac arrhythmia or significant cardiac disease, a history of cerebrovascular disease, seizures, asthma or had an autoimmune disease. Patients who had undergone major surgery within 3 weeks received chemo-, radio- or immunotherapy, including immunosuppressive therapy or had participated in another study within 4 weeks of treatment were excluded. No patient with known seropositivity for HIV or hepatitis B/C or with a known hypersensitivity to murine proteins was included. Following recruitment and within 21 days of commencing the treatment, each patient underwent a physical examination including a 12-lead electrocardiogram and a full medical history taken. The sample size was set as 45 evaluable patients using a Fleming's one sample, two-stage testing procedure for phase II studies.

### Study drug

ABR-214936 (5T4FabV13SEA_D227A_) is a 73 kDa fusion protein consisting of an engineered 5T4 Fab moiety genetically fused via the C terminus of the heavy chain to SEA_D227A_, produced in *Escherichia coli* (Forsberg *et al*, 2001). The drug compound was manufactured at Pharmacia's GMP pilot production facility in Stockholm Sweden, formulated and packed at their facility in Nerviano, Italy. It was shipped directly to the study centres on dry ice with temperature being monitored.

### Treatment plan

Patients were treated with four consecutive daily 3-h infusions of ABR-214936 at an individualised dose; a second treatment cycle was given 4–6 weeks later. Patients were given individualised doses dependent on their anti-SEA antibody titre at baseline and day 28 for treatment cycles 1 and 2 respectively, according to the schedule in Table 1. To mitigate the hypotensive effects of treatment, patients were hydrated with 1 l of saline given over 2 h before commencing the infusion; in addition, patients were treated prophylactically for fever with acetaminophen. Dose reduction was undertaken if the patient experienced multiple drug-related adverse events (AEs) at the time of administration. Thus, within the first cycle the second infusion was given at 75% of the calculated dose and if symptoms continued further reduced to 50% for subsequent days. Treatment was halted and the patient withdrawn from the study if the AE persisted at this lower dose.

### Safety and efficacy evaluation

Patient vital signs were monitored at regular intervals throughout treatment; blood samples taken before and after each infusion were used to follow changes in blood chemistry, immune cell populations, pharmacokinetics and the quantification of circulating cytokines as described previously (Giantonio *et al*, 1997). Adverse events during the study were recorded using the NCI common toxicity criteria version 2, which was current during the period of the study.

Fab-specific human anti-mouse (HAMA) titres, at baseline and following each cycle of treatment were determined by enzyme-linked immunosorbent assay (ELISA) (Immunomedics, Warren, NJ, USA). As a potential correlate for early-onset AEs systemic levels of IL-2 were measured by ELISA (Diaclone, Besançon, France) 2 h after cessation of infusion on the first 2 days of treatment during the first cycle.

Following treatment, at days 56 and 112, previously identified target lesions were assessed according to the standard response evaluation criteria in solid tumours (RECIST). Patients exhibiting reduction in tumour size at day 56 were rescanned on day 84 for confirmation.

Survival analysis based on date of death or date known alive in May 2005 was conducted with a minimum follow-up of 26 months. The 43 treated patients were divided into two groups depending on their individual drug exposure, that is, total dose received during cycle 1 (ng kg^−1^) divided by anti-SEA antibody titre (pmol ml^−1^); those above the median value (15.3) were defined as the high-exposure group (*N*=22). Patients were also grouped according to their ability to sustain a systemic IL-2 response to treatment; those with IL-2 levels above the median (4.0 ng ml^−1^) following the second infusion of the first treatment cycle were defined as high IL-2 responders (*N*=21).

Each patient was classified as high, intermediate or low risk using the methods described by Motzer (Motzer, 2003; Motzer *et al*, 2004) for untreated or previously treated patients as appropriate. Survival for a matched control group was extrapolated from published survival curves. The percentiles of the published curves were used as matched patients and the 43 patients were compared to a Kaplan–Meier curve of reference patients with the same Motzer's risk. The simulated patients were not used in any statistical test.

The influence of exposure, risk, anti-SEA, IL-2, lactate dehydrogenase (LDH), body weight, performance status and sex were tested with Cox models in SAS (PROC PHREG, SAS Institute Inc., Cary, NC, USA). A univariate analysis was performed to select important covariates. Identified covariates were used in multivariate analyses to determine the treatment effect after adjusting for risk factors.

## RESULTS

### Patient characteristics and treatment

A total of 43 Caucasian patients with a confirmed diagnosis of RCC were treated. Of these, 35 were males and eight females; the mean age was 57.6 (26–76) years; full details are outlined in Table 1. All patients had metastatic disease distributed as lung (*n*=29), lymph node (*n*=19), liver (*n*=4) and other sites including bone and soft tissues (*n*=10). Thirty-four patients had undergone resection of their primary tumour as initial treatment and a further five had had palliative nephrectomy. Seven had palliative surgery to remove metastases and one to relieve pressure on the spine. Thirty-one patients had received previous systemic treatment; chemotherapy and or immunotherapy, predominantly IFNs; of these, five patients also received radiotherapy and three patients received only radiotherapy. The median time between last previous systemic therapy and treatment with ABR-214936 was 19 weeks (range 4–74 weeks). According to the prognostic categories defined by Motzer *et al* (2004; Motzer, 2003), there were four high; 24 intermediate and 15 low-risk patients.

A total of 40 patients received two treatment cycles, and were evaluable for efficacy by computed tomography (CT) scan; of these, five patients received three cycles and a single patient received four cycles of treatment. For evaluation of survival, all 43 patients were included in the analysis.

### Safety and immunological response

Haematology, biochemistry and urinalysis gave some results outside the normal ranges, but none was considered clinically significant. During the first cycle, circulating white blood cells in particular, monocytes and lymphocytes were transiently reduced, but these changes were less pronounced after the successive infusions and not observed during the second cycle. There was a daily transient increase in temperature of 0.5–1°C, peaking at 8 h and lessening on each day. A drop in systolic and diastolic blood pressure of 5–20 mm Hg was seen during the infusions of cycle one.

Generally, the drug was well tolerated; 30 patients experienced treatment-related AEs during either cycle of treatment, the majority of which were mild or moderate. During cycle 1, 14 patients experienced AEs that resulted in a dose reduction or delay; these were typically either multiple grade 1 reactions or grade 2 pyrexia combined with grade 2 hypotension. In two cases, cessation of treatment occurred, both due to grade 3 hypotension. Only one patient required dose reduction in cycle two due to multiple grade 1 AEs. In all cases, the AEs were easily managed and resolved within 24 h of onset. The most frequent AEs experienced in cycle 1 were pyrexia (22), rigour (13), lethargy or fatigue (14), nausea or vomiting (14), hypotension (10) and hypertension (three). During the second cycle, the frequency of AEs was reduced compared to cycle 1, lethargy being the most common symptom, occurring in nine patients. Most of the patients had increased systemic IL-2 levels 5 h after the start of the first and second infusions of the first cycle.

Anti-SEA titres determined at each study visit (Table 2) were similar to previous studies (Cheng *et al*, 2004). There was a 536-fold increase in median titre following the first cycle but this was not boosted by further drug exposure and generally declined after the second cycle. Notably, four patients did not show increased anti-SEA titres after the first therapy cycle. No patients had measurable HAMA before commencing treatment (Table 2). Following one cycle of treatment, nine of 41 patients (22%) had a measurable HAMA titre above the limit of quantitation for the assay (⩾0.24 pmol ml^−1^). After the second cycle of treatment, this had risen to 22 of 26 patients (85%).

### Efficacy

Primary efficacy was evaluated using CT scan according to the RECIST criteria. At day 56, one patient showed a partial response (PR), whereas the largest proportion, 27 patients, showed stable disease (SD) and 12 had progressed (PD). Three patients were not evaluated as they had either withdrawn consent (*N*=2) or not been able to undergo CT-scan (*N*=1). At day 112, one patient continued to show PR, 16 SD, 21 had progressive disease and two further patients were nonevaluable.

**Figure 1:**
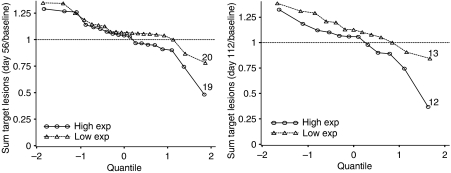
Change in tumour volume at day 56 and day 112 relative to baseline, measured by CT scan. The rate of growth for the high drug exposure group is slower than for the low exposure group. The number of patients in each group is indicated.

The changes in size of target lesions at days 56 and 112, relative to baseline measurements are illustrated in Figure 1. At both assessment times, patients with high drug exposure exhibited a slower rate of tumour growth or greater reduction in size than the low-exposure group. At day 56, in addition to the one clear response, a further nine patients showed a 10–25% reduction in tumour volume. Of these, seven had high drug exposure compared with only two low exposure patients. Similarly, at day 112 of the six patients showing a reduction in tumour volume, four were in the high and two in the low exposure groups. A 25% reduction in one patient at day 56 was sustained at day 112.

**Figure 2:**
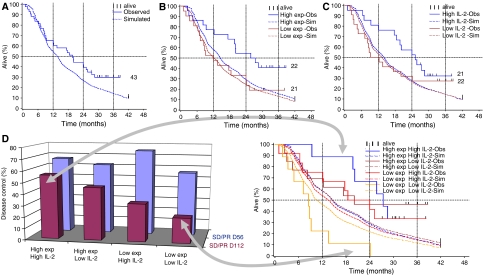
Survival for the patients treated with ABR-214936 was compared to a matched simulated control population with equivalent Motzer's characteristics. Overall, median survival (**A**) was 19.7 months *vs* 13.7 for the controls and 2-year survival was 42 *vs* 27%. As a function of drug exposure (**B**), median survival for the high-exposure group was 26.6 *vs* 12.1 months for the low exposure group (controls 14.5 and 12.2 months). As a function of IL-2 after the second infusion (**C**), median survival for the high exposure group was 25.2 *vs* 10.2 months (controls 14.0 and 13.3 months). Comparing drug exposure, disease control and IL-2 response to survival (**D**) shows that patients with high IL-2 after the second infusion and high exposure are more likely to have disease control at day 112 and the longest survival. The number of patients in each group is indicated.

Median time to progression was 4.0 months and median survival was 19.7 months with 13 patients still alive at the time of writing, a 2-year survival of 42%. Figure 2A shows patient survival with a minimum follow-up of 26 months plotted against a simulated patient population with matching risk factors as defined by Motzer's score. In our patient group, the median survival of 19.7 months compares favourably with the expected survival of 13.7 months. Expected median survival for our patients in the high, intermediate and low risk categories were 5.1, 11.3 and 21.8 months, respectively, by comparison, their observed survival was 3.1, 15.6 and 25.8. Both the low and intermediate risk patients show an increase in survival time compared to published expectation.

From a toxicity perspective, there was a correlation between MTD and anti-SEA levels; thus for this product, drug exposure is defined as dose divided by anti-SEA levels. Patients receiving higher exposure show a survival advantage compared to the low exposure group (Figure 2B); 26.6 months *vs* 12.1 months, and have a greater proportion of long-term survivors (nine *vs* four). Expected survival for the matched controls was 14.5 and 12.2 months, respectively.

Serum IL-2 was measured on days 1 and 2 of the first treatment cycle (Table 2). Patients categorised as high-IL-2 responders on day 2 (⩾4 pg ml^−1^) have a better outcome than low responders (Figure 2C). No correlation between day 1 IL-2 levels and survival was observed. A univariate analysis indicated five significant (*P*<0.05) factors associated with increased survival. Those were favourable Motzer's risks, normal LDH levels, high weight, high exposure and high-IL-2 levels after the second infusion. A bivariate analysis, correcting for Motzer's risks, supported the univariate analysis and both exposure and IL-2 after the second infusion reduced the risk of dying by about 50%. Finally, a multivariate analysis of IL-2 after the second infusion, exposure and Motzer's risks was a very good model (*n*=43). In this, the risk of dying was reduced by about 48% (95% CI: 20–66%) for each unit in the log scale for IL-2 (1 unit increase in the e-logarithm corresponds to a 2.7 times higher value). The corresponding reduction in the risk of dying for patients with high exposure compared to those with a low exposure was 71% (95% CI: 31–88%). Analyses with the covariate ‘Motzer's risks’ replaced with ‘elevated LDH’ as well as addition of body weight as covariate supported those findings. Thus, drug exposure and sustained IL-2 levels after second infusion act additively.

The connection between disease control, survival, exposure and IL-2 response on day 2 in the first cycle is illustrated in Figure 2D. The best disease control and survival was seen in the group having both high exposure and high IL-2 and the poorest with low exposure and low IL-2. However, both the high exposure/low IL-2 and low exposure/high IL-2 groups also show evidence of disease control and improved survival. There is a correlation between exposure and disease control at day 112, which affects the long-term survival.

**Figure 3:**
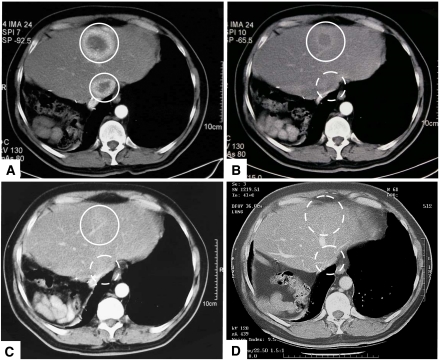
CT scans of the patient who showed sustained PR during the trial: At baseline (**A**), day 84 (**B**), 12 (**C**) and 36 (**D**) months post-treatment. Resolution of one liver metastasis is evident by day 84 with significant ablation of the second, which also eventually resolves. The patient is a long-term survivor at ++36 months and considered in full remission.

The patient who showed PR received an exposure during the first cycle, five times greater than the median. This patient experienced multiple grade 1 AEs in the first cycle, but later received three further cycles at full dose. As can be seen in Figure 3 there was a dramatic reduction of the metastatic lesions in the liver, there was also shrinkage of a local recurrence. The patient has survived for more than 36 months since treatment and no longer has any evidence of active disease at any of the sites; however, is classified as a PR based on the response at day 112. At 12-months follow-up (Figure 3C) there remained an indeterminate CT shadow at one lesion site in the liver, but by 19 months this had completely resolved and both sites remain clear at 36 months (Figure 3D). Although, spontaneous regression is known in RCC, it is relatively rare and there is no reliable predictor for when this is likely to occur. In our experience the median duration of spontaneous remission is approximately 7 months; consequently the long-term disease-free survival of this patient is strong evidence for drug-induced remission. A second patient, with both liver and skin metastases, classified as PD owing to progression of the skin metastasis, showed complete resolution of the liver metastasis after treatment and following the surgical removal of the skin metastasis, is considered to be in complete remission. Interestingly, these two patients showing the best clinical outcome also had the highest IL-2 responses on day 2 of treatment (17.8 and 87.2 pmol ml^−1^).

Four patients were evaluated by 18-fluorodeoxyglucose -positron emission tomography (FDG-PET) at day 56, all showed SD by CT at day 112; however, one showed a response using PET, but only marginal shrinkage using CT scan. Two patients were confirmed as SD and one showed progression.

## DISCUSSION

In this paper, we present the first data from RCC patients treated with a tumour-targeted superantigen. Despite limited patient numbers and no randomized control group, the prolonged survival suggests treatment benefit, based on two-independent comparisons. First, our patients had a median survival of 19.7 months, considerably longer than the 13.7 months survival (Figure 2A) in matched controls predicted from the extensive analyses by Motzer (2003; Motzer *et al*, 2004). It is also longer than the 8–15 months survival seen with other therapeutic agents assessed in recent studies in similar patient populations (Mekhail *et al*, 2005). These studies have included immunotherapy with IFN-*α* or IL-2 alone or in combination with established chemotherapy (Motzer and Russo, 2000); thalidomide (Clark *et al*, 2004), 5-FU (O'Brien *et al*, 2004; Rathmell *et al*, 2004), chemotherapy; gemcitabine+oxaplatin (Porta *et al*, 2004), gemcitabine+capecitabine (Waters *et al*, 2004) and experimental agents such as Gefitinib (Dawson *et al*, 2004).

Second there is a relationship, although not significant (*P*=0.098), between drug exposure and a reduction in the rate of tumour growth (Figure 1). Furthermore, a relationship exists between drug exposure and survival (Figure 2B), where high exposure patients lived twice as long as low exposure patients. To simulate the expected survival times of matched controls we used prognostic factors described previously (Motzer, 2003; Motzer *et al*, 2004) to group them according to risk and whether they had PD on previous therapy (i.e. IFN-*α*).

Despite the long survival times, the response rate was rather modest. However, a large fraction of the patients had SD lasting for at least 4 months, which is comparable or better than concurrent studies in similar patients with other experimental agents (Dawson *et al*, 2004; Porta *et al*, 2004; Rathmell *et al*, 2004). Patients who received a high exposure were more likely to have disease control at day 112 and a long survival (Figure 2D). It is clear that several novel anticancer therapies give clinically beneficial effects without giving objective responses on CT scan; however, FDG-PET analysis may be a useful tool to gain further information. Of four patients who showed SD by CT, one showed a response using PET but the shrinkage using CT scan was only 13%. This patient from the intermediate risk group is still alive after more than 30 months. Another patient showed progression using PET but only a 5% tumour increase using CT scan. This patient in the high-risk group lived 9 months. Thus, PET may be a better tool than CT for the evaluation of tumour responses following ABR-214936 therapy. Interestingly, two patients with liver metastases, associated with poor prognosis in RCC (Mekhail *et al*, 2005), showed complete eradication of these metastases after ABR-214936 treatment. In a previous trial using another antibody targeted superantigen, eradication of liver metastases was also observed (Alpaugh *et al*, 1998a) and it is therefore possible that the liver associated disease is particularly sensitive to superantigen therapy.

The mechanism of action of the TTS involves targeting of cytotoxic-T cells to the tumour tissue. However, on the basis of preclinical observations (Rosendahl *et al*, 1996; Litton *et al*, 1997) this is achieved by a stepwise process. Initially, the systemic activation and expansion of the superantigen reactive T cells, then localisation of the activated T cells to the tumour followed by T cell-mediated killing of the tumour cells. This multistep process necessitates the repeated administration of the fusion protein.

In previous studies (Alpaugh *et al*, 1998b; Cheng *et al*, 2004), the occurrence of high-systemic levels of IL-2 correlated with toxicity. In the present study, post-infusion IL-2 levels were measured on the first 2 days of treatment as this coincided with the majority of AEs. This analysis showed that 68 of the 74 rapid-onset AEs occurred in the group of patients with IL-2 above the median on day 1 of treatment (>9.2 pg ml^−1^), confirming the previously observed correlation. However, in this study we have been able to go further in the understanding of the relationship between systemic IL-2 and the mechanism of action of TTS therapy by describing a direct relationship between a patient's ability to sustain an IL-2 response following the second infusion of ABR-214936 and improved survival. Patients mounting a sustained IL-2 response had a median survival more than twice that of nonresponders (Figure 2C). This difference was not a result of covariation with performance status or Motzer's risks (data not shown). Furthermore, the IL-2 levels seen were lower than those achieved during systemic IL-2 therapy, ruling out that the survival benefits were primarily caused by the IL-2. Notably, there was no correlation between the IL-2 levels induced after the first infusion and survival, indicating that it is the sustained T-cell activation that is necessary for therapy as suggested by preclinical findings (Rosendahl *et al*, 1996).

A phase I trial of ABR-214936 performed in patients with NSCLC (Cheng *et al*, 2004) had the primary end point of safety, but CT scan and survival were also investigated. Interestingly, several of the observations in our phase II study were seen in the phase I trial. For instance, despite no objective responses being recorded, a large proportion of the patients showed SD (Cheng *et al*, 2004). Also, there was a correlation between high exposure and longer survival (unpublished observation).

The rate of induction of HAMA observed in this study following one and two treatment cycles was 22 and 85%, respectively (⩾ 0.24 pmol ml^−1^), comparable to the frequency reported for other antibodies used in treatment or imaging of solid tumours (Mirick *et al*, 2004). However, the magnitude of this reaction is significantly lower than those reported previously, by a factor of between 9.5 and 265 times depending on the type of assay used (Kricka *et al*, 1993; Mirick *et al*, 2004). Thus, the induction of a HAMA is unlikely to have any significant contribution to the biodistribution or toxicity of the ABR-214936 when compared to the level of pre-existing anti-SEA seen in most patients (Table 2).

The side effects recorded in this phase II trial were generally mild and easily managed. In most cases they involved fever, nausea and rigours. This is similar to the side effects observed in the phase I trial which suggest that the product has a similar toxicological profile in RCC and NSCLC patients. However, even though the side effects were mild, there is a correlation between MTD and the patients' anti-superantigen antibody titre. Patients with relatively higher antibody titres tolerated higher doses of ABR-214936 with identical side effect profile as compared to patients with lower titres receiving lower doses. Patients were grouped according to their anti-SEA titres; doses received varied between 60 ng kg^−1^ day^−1^ for the lowest titre group and 1200 ng ^−1^kg^−1^ day^−1^ for the highest. From a safety point of view, this appears to have been a good approach, but a consequence was that the patients received different exposures (ratio of administrated dose of ABR-214936 divided by anti-SEA). As there was a strong survival benefit for patients receiving a higher ratio, this dose group strategy has to be modified to make sure that all future patients are optimally dosed. Toxicity during the second cycle was considerably lower than during the first and only one patient required dose reduction. This is because, relative to the first cycle, most patients may have been under-dosed owing to their increased anti-SEA titre.

Although it is possible to measure the patients' titres of anti-SEA antibodies before therapy, it is not a convenient approach. Therefore, in parallel to the clinical trials of ABR-214936, novel superantigen variants with low reactivity to human anti-SEA antibodies have been designed (Erlandsson *et al*, 2003). The removal of the link between anti-SEA titre and dose should allow every patient to receive an optimal dose and thus improve the efficacy of treatment. One of those superantigen variants, SEA/E-120 fused with the 5T4Fab moiety is the optimised TTS ABR-217620 currently in phase I dose escalation studies in patients with NSCLC, RCC and pancreatic cancers.

One future direction for the development of RCC therapy currently under investigation is targeted antiangiogenic agents (de Gramont and Van Cutsem, 2005; Patel *et al*, 2006). This includes receptor tyrosine kinase inhibitors and antibodies directed against growth factors such as vascular endothelial growth factor, either singly or in combination. So far, the best objective responses have been seen with sunitnib (40%) and combination therapy of bevacizumab and erlotinib (25%) with median disease-free periods of 8.7 and 11.1 months, respectively. The 2-year survival with sunitinib is 40%, which is comparable to the 42% 2-year survival for patients treated with ABR-214936 in this study (Motzer *et al*, 2006; Patel *et al*, 2006). However, the majority of patients still progress following these antiangiogenic therapies; thus, although it is an improvement on the response rate achieved with IFN-*α* or IL-2 there is still a requirement for further development of novel therapies.

## Figures and Tables

**Table 1:** Patient characteristics, dosing and response to treatment

**Characteristic**	**No**	**%**	**Characteristic**		**No**	**%**
Total	43	100	Male		35	81
						
*Age, years*			*Previous therapy*			
Median	57.6	—	Median no of treatments		1	—
Range	26–76	—	Range		0–4	—
						
			Radiation		8	19
*Karnofsky performance status* (%)			IL-2		13	30
100	8	19	IFN		25	58
90	28	65	Chemotherapy		8	19
80	7	16	None		9	21
						
*Motzer risk factor*			*Patients with metastatic disease*		43	100
0=low	15	35	Lung		29	67
1=intermediate	24	56	Liver		4	9
=high	4	9	Lymph nodes		19	44
			Other (including bone)		10	23
*Histology*						
Clear cell	27	63	*Dosing with ABR-214936 (cycle 1)*			
Papillary	1	2	*α*-SEA titre pmol ml^−1^	Dose ng kg^−1^		
Ductal	2	5	<40	60	1	2
Unspecified	13	30	41–50	100	7	16
			51–90	300	15	35
*Drug exposure (Dose:α-SEA)*			91–150	500	6	14
High (>15)	22	51	151–300	800	9	21
Low (<15)	21	49	>301	1200	5	12
						
*Previous surgery*			*Best overall response (day 56)*			
Nephrectomy	34	86	PR		1	2
Removal of metastases	7	16	SD		27	63
None	6	14	PD		12	28
			N/A		3	7

IL-2=interleukin-2; IFN=interferon; SD=stable disease; PD=progressed; PR=partial response.

**Table 2:** Physiological responses to ABR-214936 treatment

	**Anti-SEA (pmol ml^−1^)**			**HAMA (pmol ml^−1^)**			**IL-2 (pg ml^−1^)**		
**Visit**	***n***	**Median**	**Range**	***n***	**Median**	**Range**	**Mean±s.d.**	**Median**	**Range**
Baseline	43	87.0	15–6400	43	0	0–0			
Day 1	43						37.8±70	9.2	0–357.7
Day 2	43						6.7±13.3	4	0–87.2
Day 28	40	46 700	79–445 000	41	0.058	0–9.36			
Day 56	38	31 600	113–241 000						
Day 112	27	25 100	175–176 000	26	1.542	0–16.4			

Anti-SEA and HAMA titres measured at baseline and after each cycle of treatment and circulating IL-2 measured on days 1 and 2 of cycle 1.
